# Oral health-related quality of life after dental treatment in patients with intellectual disability

**DOI:** 10.4317/medoral.23549

**Published:** 2020-07-19

**Authors:** Virginia Rollon-Ugalde, Jose Antonio Coello-Suanzes, Ana Maria Lopez-Jimenez, Javier Herce-Lopez, Pilar Toledano-Valero, Javier Montero-Martin, Pedro Infante-Cossio, Angel Rollon-Mayordomo

**Affiliations:** 1 Department of Oral and Maxillofacial Surgery, Virgen Macarena University Hospital, Seville, Spain; 2 Department of Experimental Psychology, Faculty of Psychology, University of Seville, Seville, Spain; 3 Department of Surgery, School of Medicine, University of Salamanca, Salamanca, Spain; 4 Department of Surgery, School of Medicine, University of Seville, Seville, Spain

## Abstract

**Background:**

The influence of dental treatment on oral health-related quality of life (OHRQOL) has rarely been evaluated in patients with intellectual disability (ID) through validated questionnaires. The aim of this study was to estimate the changes on OHRQOL in patients with ID after the implementation of an institutional dental treatment program under general anesthesia using the Franciscan Hospital for Children Oral Health-Related Quality of Life questionnaire (FHCOHRQOL-Q).

**Material and Methods:**

A prospective longitudinal study was conducted on 85 patients (mean age=24.85 years) classified according to DSM-V whose parents/caregivers completed the FHC-OHRQOL-Q. We analyzed the changes in the questionnaire’s overall score and its dimensions from pre-treatment to 12-months of follow-up, considering effect sizes and minimal important differences estimated by the standard measurement error. The impact of clinical and therapeutic factors was evaluated using univariate and multiple linear regression analysis (*p*<0.05).

**Results:**

Significant improvement of OHRQOL was found after dental treatment in oral symptoms (*p*0.001), daily life problems (*p*=0.018), parent’s perceptions (*p*=0.013) and FHCOHRQOL-Q´s overall score (*p*=0.001). OHRQOL changes exhibited an intermediate magnitude (0.38-0.21) as estimated by effect sizes. Changes in oral symptoms showed positive correlation with DMFT index (*r*=0.375, *p*=0.002), decayed teeth (*r*=0.244, *p*=0.036), dental extractions (*r*=0.424, *p*<0.001) and number of treatments (*r*=0.255, *p*=0.019). The improvement was greater in patients with 4 decayed teeth (*p*=0.049) and undergoing 2 dental extractions (*p*=0.002). Multiple regression analysis demonstrated that dental extractions (*p*<0.001) and DMFT index (*p*=0.028) were significantly related to oral symptom improvement.

**Conclusions:**

Dental treatment under general anesthesia showed a positive effect on the overall FHC-OHRQOL-Q score and most of its dimensions. At 12-months of follow-up, the improvement of oral symptoms was significantly associated with DMFT index, decayed teeth, dental extractions and number of treatments. In our clinical setting, the implementation of a dental treatment program enhanced the OHRQOL of patients with ID.

** Key words:**Oral health-related quality of life, intellectual disability, general anesthesia, special needs, dental treatment, Franciscan Hospital for Children Oral Health-Related Quality of Life questionnaire.

## Introduction

Oral health-related quality of life (OHRQOL) is a multidimensional construct that has become a very important parameter to assess how oral health impacts on daily function, well-being and social interaction of people with special health care needs (SHCN). People with SHCN, including those with intellectual disability (ID), often show poor oral health due to various developmental disorders and/or cognitive disabilities, along with limitations to tolerate routine treatment at the dental clinic (1). In addition, general anesthesia (GA) is required in many of them to provide safe dental treatment given their persistent inability to cooperate, the severity of their dental problems, and the complex and extensive procedures they often need (2,3).

The measurement of OHRQOL in patients with ID is not only subjective but also very difficult to estimate due to their communication deficiencies and, therefore, can only be indirectly assessed through an instrument that must be handled by their parents and caregivers. However, despite the unquestionable significance of measuring OHRQOL in dental clinical practice, there are few published studies that have focused on the influence of dental treatment in patients with ID with validated quality of life (QOL) questionnaires in the short- and mid-term (4,5).

The Franciscan Hospital for Children Oral Health-Related Quality of Life questionnaire (FHCOHRQOL-Q) (6) is a tool designed specifically for parents and caregivers of individuals with ID to detect their dental treatment needs, which was previously validated to Spanish by our team (7). The FHCOHRQOL-Q adapted to Spanish proved to be a reliable and valid instrument to evaluate the impact of OHRQOL in daily clinical practice. In the present study, we aimed to estimate the changes on OHRQOL in a group of mainly adult patients with ID after undergoing dental treatment under GA, using the Spanish-adapted FHCOHRQOL-Q. We evaluated the influence of dental treatment and the impact of clinical and therapeutic factors on OHRQOL from pretreatment to 12 months of follow-up.

## Material and Methods

-Study design

A longitudinal prospective study was conducted in the Department of Oral and Maxillofacial Surgery, Virgen de Macarena University Hospital, Seville, Spain. The study included all patients with ID referred to receive dental treatment under GA, from June 2012 to June 2016, who came accompanied by their parents or responsible caregivers. Before the start of the study, parents and caregivers were given information about the study characteristics and signed informed consent. The present report followed the Strobe recommendations and was carried out in accordance with the guidelines proposed in the World Medical Association‘s Declaration of Helsinki (8). The study was approved by the Ethical Committee of the Hospital (file number: 0871-N-14).

- Study population and procedures

**Figure 1:**
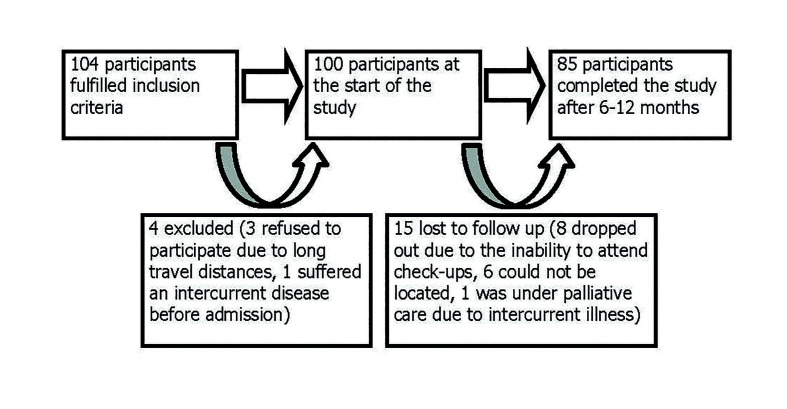
Participants recruitment in the study.

Patients were classified according to the American Psychiatric Association Diagnostic and Statistical Manual of Mental Disorders (DMS-V) as suffering: 1) global development retardation, 2) deep intellectual development disorder, 3) non-specific intellectual disorder, 4) schizophrenia, 5) rare illness, or 6) Down syndrome. The recruitment process is depicted in Fig. 1. Of 104 consecutive patients who initially met the pre-inclusion criteria, 4 were excluded for different reasons. Among the 100 individuals enrolled at the start of the study, 85 completed it while 15 were lost to follow-up.

The procedures consisted of 1) a QOL survey prior to the intervention, 2) an oral examination and dental treatment performed in a single operative session, and 3) a postoperative QOL survey at the check-up visit. On the same day of the intervention, parents/caregivers filled out the Spanish-adapted FHC-OHRQOL-Q in the presence of an interviewer. Under GA, two researchers performed a complete oral examination and recorded the following WHO Criteria (9): dental status, caries, periodontal status, teeth loss and DMFT index (decay-missing-filled teeth). Dental treatments consisted of dental extractions, restorative treatments with fillings/endodontics, and periodontal treatments including scaling and root planing. Between 6 and 12 months after the treatment, the patient was checked at the outpatient clinic and parents/caregivers filled out the FHC-OHRQOL-Q again in the presence of an interviewer who was unaware of the previously performed dental treatment.

- Questionnaire

The FHC-OHRQOL-Q is an instrument designed to be completed by parents and caregivers, which consists of 41 items distributed in four dimensions: oral symptoms (D1), daily life problems (D2) and parental concerns (D3). The answers are scored from 0 (never) to 5 (always). Parents' perception (D4) is assessed through a visual analog scale (VAS, 0-10 cm). The score of each dimension was obtained from the mean of its items. The overall score was obtained by adding D1, D2 and D3 values. Higher scores indicate worse OHRQOL.

- Statistical analysis

Data were analyzed using the statistical package SPSS V.22 (Statistical Package for the Social Sciences, Inc., Chicago, IL, USA). Quantitative variables were presented as mean (M), median (Md), range and standard deviation (SD). Effect sizes were used to calculate the changes through the mean differences of the pre-treatment and post-treatment result. Values of <0.2, 0.3-0.7, or >0.8 corresponded to a small, intermediate and large effect sizes, respectively (10). Minimal clinically important differences were based on the determination of the standard error of measurement and calculated by the equation SD*√(1-reliability), where the reliability is equal to the Cronbach's alpha coefficient (11-13). Internal reliability was assessed according to Cronbach's alpha coefficient of the preoperative value. The primary outcome of this study was “oral symptoms” (D1). The sample size was calculated to detect a 25% reduction in D1, which is equal to an effect size value of 0.3, taking into account a baseline SD of 0.85. With an alpha error of 0.05 and a power of 0.8, it was determined that a sample of 93 subjects would be needed.

Age, DMFT index, number of decayed teeth, periodontal status, and number of treatments were related to each dimension change by using correlation coefficients. Statistical differences were identified among groups established from the median of the clinical and therapeutic variables. Normal distribution of values was verified by Kolmogorov test. Student t test, U Mann Whitney, Wilcoxon and Pearson correlation coefficient were used to analyze the statistical significance, which was considered at a value of *p*<0.05. Multiple linear regression analysis was performed to quantify the effect of each variable on D1, D2, D3 and overall score changes. The variables introduced were those that were correlated in the univariate analysis with a level of significance of *p*<0.15.

## Results

Of 100 patients who participated in the study, 59 were males and 41 females with a mean age of 24 years (SD=13.1, range=4-71 years). A total of 85 patients, 49 (57.6%) males and 36 (42.4%) females with a mean age of 24.85 years (SD=14.18, range=7-71 years) completed the study. 15 patients were lost to follow-up, 10 (66.7%) males and 5 (33.3%) females with a mean age of 21.43 years (SD=14.48, range=4-70). Both the 85 participants and the 15 patients lost to follow-up were homogeneous groups insomuch that no significant differences were found in the distribution of the numerical and non-numerical variables.

The causes of ID in the 85 participants were psychomotor retardation [22], cerebral palsy [21], developmental disorder [19], rare diseases [15], Down syndrome [6], and psychiatric disorder [2]. 70 of the caregivers were family members (62 mothers, 2 fathers, 4 both, 1 grandmother and 1 brother) while 15 patients lived in an institution, so their caregivers were the responsible workers of the institution. The parents/caregivers filled out the FHC-OHRQOL-Q at the post-treatment check-up visit between 6 and 12 months (Md=9). 9 (10.5%) questionnaires were completed by an institutional caregiver that was not the same as the initial visit, although both were the usual responsible caregivers of these patients.

Oral examination at baseline and dental procedures performed per patient are shown in Table 1. Oral examination showed a mean DMFT index of 5 (SD=4.13) and the mean of decayed teeth per patient was 4.99 (SD=3.31). In total, 445 dental procedures were performed consisting of 217 fillings, 181 dental extractions, 44 scaling and root planing and 3 endodontic treatments.

The percentage of non-answered items in each dimension of the questionnaire ranged between 0.66% for D1 and 0.2% for D2. All the dimensions exhibited a normal distribution, with the exception of D2. Table 2 shows the mean differences of the dimensions and overall score at pre-treatment and follow-up. All mean differences showed a positive significant improvement (*p*<0.05) except D3. The magnitude of changes measured by effect sizes was considered intermediate and ranged between 0.38 in D1 and 0.21 in D3. The percentage of patients who improved and exceeded a standard error of measurement value ranged from 55.3% in D1 and 36.5% in D2.

**Table 1:**
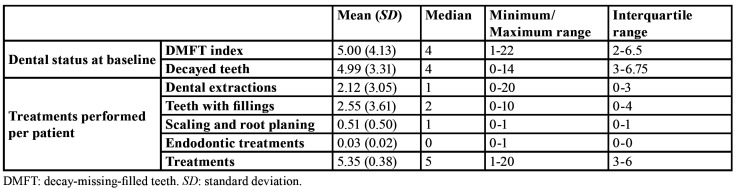
Dental status at baseline and treatments performed per patient (n=85).

**Table 2:**
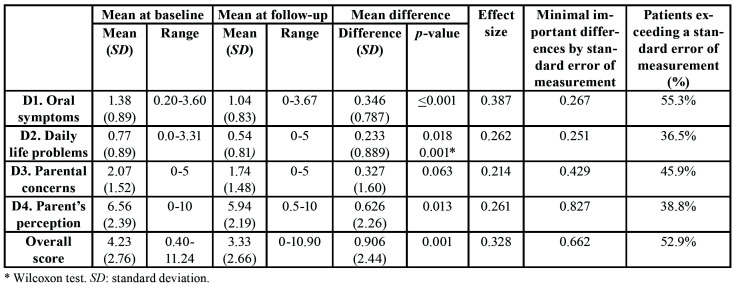
Mean differences of the dimensions and overall score at baseline and follow-up (n=85).

Table 3 presents Pearson´s correlation coefficients and significance level of post-treatment changes in relation to the clinical and therapeutic variables. The improvement in D1 exhibited a significant positive correlation with DMFT index (r=0.375, *p*=0.002), number of decayed teeth (r=0.244, *p*=0.036), number of dental extractions (r=0.424, *p*<0.001) and number of treatments (r=0.255, *p*=0.019). The number of filled teeth correlated negatively with the change in D3 (r= -0.324, *p*=0.003) and with the change in overall score (r= -0.251, *p*=0.021). Endodontic treatments were not analyzed because of their low number.

Table 4 shows the mean differences of the dimensions and overall score when comparing pre- and post-treatment values according to the cut-off points established by the medians of the clinical and therapeutic variables. The change in D1 exhibited significant positive difference in patients with >4 decayed teeth (*p*=0.049) and undergoing >2 dental extractions (*p*=0.002). The change in D3 showed significant negative difference in patients undergoing >6 treatments (*p*=0.037) and >3 filled teeth (*p*=0.001). The change in overall score showed significant negative difference in patients with >3 filled teeth (*p*=0.005).

**Table 3:**
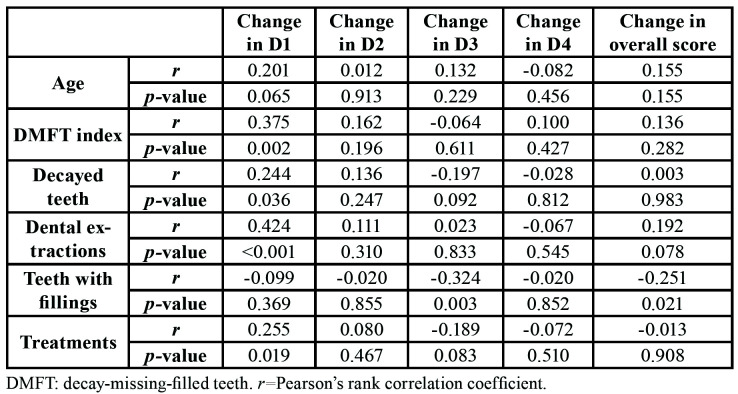
Pearson´s coefficients and significance level of post-treatment changes of the dimensions and overall score in relation to clinical and therapeutic variables (n=85).

**Table 4:**
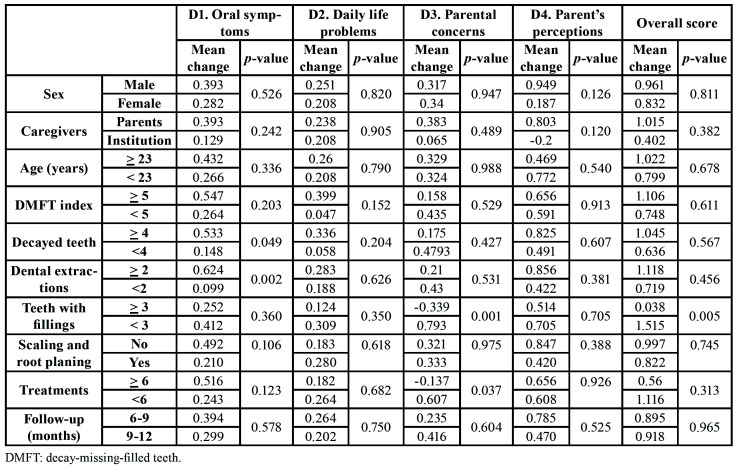
Change in means of the dimensions and overall score, prior and after dental treatment, according to the medians of clinical and therapeutic variables (n=85).

The predictive variables used to perform the model of multiple linear regression analysis were number of dental extractions, DMFT index, and number of decayed teeth, filled teeth and treatments. The model of analysis revealed that the change in D1 obtained an R2 of 0.319, with number of extractions (*p*<0.001) and DMFT index (*p*=0.028) being the significant variables. The values ​​of D1 improved by 18.7% (CI=9.2%-28.2%) for each dental extraction, and by 7% (CI=0.8%-13.2%) for each unit of DMFT index increase. The analysis of D3 and overall score obtained an R2 of 0.13 and 0.09, respectively, with the number of filled teeth being the significant variable for both D3 (*p*=0.007) and overall score (*p*=0.032). The values ​​of D3 and overall score worsened by 27% (CI=8%-47%) and 21% (CI=1.9%-41.4%), respectively, for each increase per unit of filled teeth. Reliability analysis with Cronbach's alpha coefficient showed values >0,88 and no items were found whose elimination would cause an increase in reliability.

## Discussion

This prospective longitudinal study describes the clinical experience of a single center over a period of 4 years, analyzing how dental treatment under GA may influence the OHRQOL of patients with ID. OHRQOL was evaluated in a sample of patients with ID whose parents/caregivers completed the FHCOHRQOL-Q prior and up to 12 months after treatment. The participants were mostly adults (M=24.85 years), considering that 75% of them were >15 years old and only 10% were <10 years old. To our knowledge, this is the first survey that examines the potential association between dental treatment and OHRQOL in this type of patients in Spain. The main finding of the present study revealed a significant improvement of the overall score and most of the dimensions of the FHCOHRQOL-Q after the implementation of an institutional dental treatment program. In general terms, dental treatment had a positive impact on the way parents and caregivers perceived the OHRQOL. The relatively high sample size (85 patients) allowed us to perform a model of multiple linear regression analysis and a correlation analysis to explore how clinical and therapeutic factors could influence the results in the mid-term (6-12 months). The loss of patients (15%) was low compared to other studies that reported rates between 0 and 47.8% (4,6,14-16). However, the homogeneity among the groups of 85 participants and the 15 lost patients ensured that the validity of our study was not affected.

There is no consensus on which questionnaire should be used to analyze changes on OHRQOL in individuals with SHCN. We used the validated and Spanish-adapted FHC-OHRQOL-Q, whose structure is similar to other specific instruments to assess oral health commonly used among children (7,15). The FHC-OHRQOL-Q can be used as a diagnostic tool to detect dental treatment needs in patients with SHCN who are difficult to explore and eventually require dental treatment more immediately. The instrument also provides excellent information on the effect that different dental treatments have on the OHRQOL, and thereby, can help make decisions about the best procedures to perform for each patient. Ultimately, the instrument has proved useful for monitoring results in the short and medium term. In all the steps, the questionnaire behaved as a simple, widely accepted and easy tool to administer to parents and caregivers.

To the best of our knowledge, few studies have evaluated the changes on OHRQOL after dental treatment under GA in patients with intellectual and developmental disabilities. Chang *et al*. (4) and Hillebrecht *et al*. (5) used the COHIP-14 and OHIP-G14 questionnaires; Song *et al*. (17) added the FIS-12 questionnaire; and Baens-Ferrer *et al*. (6) described the FHC-OHRQOL-Q; but only the first two studies considered the effect of dental treatment on a population of adult patients with ID. As a whole, these studies reported that OHRQOL improved significantly after dental treatment compared to baseline. Our findings with the FHC-OHRQOL-Q supported these data previously reported as we found significant changes in the overall score and in D1, D2, D4, with the exception of D3. This exception could be due to the fact that our patients were mostly adults who had worst D3 baseline value, reflecting other problems and concerns not solved with dental treatments. The characteristics of the patients and parents/caregivers and the treatments performed in our study were very similar to the previous ones, showing only slight differences depending on causes of the ID, age and oral health of the sample, and types of treatment included.

Effect sizes were used to evaluate the magnitude of the post-treatment result achieved (18-19). The QOL changes estimated by effect sizes revealed an intermediate magnitude in our study, with the high values in D1 (0.38) and overall score (0.33). This intermediate magnitude can be explained by the chronic and severe disease that our patients had, the higher expectations of their parents/caregivers and the worst baseline score in the OHRQOL dimensions (20-22). Chang *et al*. (4) described a large magnitude in oral health and overall QOL and a small in the physical dimension and Family Impact Scale (FIS). Other studies in children showed a large magnitude in overall QOL and oral dimension, and an intermediate and small in other dimensions after 4 weeks of treatment (14,16,19).

To facilitate the interpretation of the results and evaluate their clinical importance, we have measured minimal important differences according to the Wyrwich´s concept (13). These values represent the minimum change required for parents and caregivers to perceive noticeable and beneficial changes in the health of patients after dental treatment. We used the standard error of measurement because its value is independent of the sample, which becomes a good indicator of intra-individual changes (23). The minimal important differences were exceeded by 55.3% of the parents/caregivers in D1 and in 52.9% in the overall score, which supported a relevant clinical improvement in the main dimensions. The dimensions that less exceeded minimal important differences were D2, D3 and D4, possibly because they reflected other problems that were difficult to manage with dental treatment. These results are consistent with other studies in children without disabilities (24,25).

To delimit the theoretical structure that supports the OHRQOL changes, we analyzed the clinical and therapeutic factors that could influence or explain our results (8). In our study, the change in D1 showed a significant correlation with DMFT index and number of decayed teeth, dental extractions and treatments, being the improvement significantly higher in patients with >4 decayed teeth and undergoing >2 dental extractions. These data indicated that the patients who improved their oral symptoms the most were those with the worst previous dental health due to lack of prior treatments and those who required more therapeutic procedures. We did not find a significant correlation with the changes in D2 and D4. D3 and overall score exhibited a significant negative correlation with filled teeth, which revealed a significant worsening in D3 related to patients undergoing >6 treatments and >3 filled teeth, and in overall score to >3 filled teeth. This deterioration can probably be related to the worst dental health status of patients and the greatest cognitive impairment, and as a result, D3 could hardly improve. We did not find differences when we analyzed age, sex or type of caregiver (parent or responsible institutional caregiver). Although age did not show a significant correlation, we found an improvement in all dimensions in patients older than 22 years, except for D4 (4).

The correlation between type of dental treatment and improvement of OHRQOL has been poorly evaluated in the literature (15). Our results showed that the procedure that had the greatest significant effect was dental extraction, which significantly improved those items related to oral symptoms (D1), although its effect was fewer in the other dimensions of the questionnaire. One study described that root canal treatment significantly improved overall QOL when measured as a dichotomous variable (4), although the rate of endodontic treatment (38.2%) was higher than that of our study (3.5%). Other studies found no relationship between dental extractions or fillings and improvement of overall QOL (4,15,24). Interestingly, scaling and root planning did not show a significant association with any dimension in our study.

In our study, the median of follow-up check-ups was 9 months (range=6-12 months). In previous studies, the cut-off points for follow-up varied between 1-4 weeks and 9 months. In the mid- and long-term, QOL can be affected by recurrence of oral disease and adaptation of patients and caregivers over time. El Batawi *et al*. (26) reported that 59% of children had new carious lesions after 2 years of treatment, and Xiao *et al*. (27) found 37% after 6 months. In our study, we did not find a significant correlation between changes in dimensions and follow-up scores, nor significant differences when comparing groups of 6-9 and 9-12 months.

In the model of multiple linear regression analysis, only dental extractions and DMFT index were significantly related to the improvement in D1, which confirmed the results of the univariate analysis. These results are consistent with the study by Hillebrecht *et al*. (5) that reported a significant correlation of OHRQOL improvement after dental extractions in patients with cognitive disability. This model of analysis only explained 13% and 9% of the variance in D3 and overall score, respectively, Figures that we consider not relevant.

The strength of our study is based on the use of a validated questionnaire, applied on the same milieu in which the authors conducted the previous validation study (7,28). The sample was homogeneous with a relatively high number of patients with ID. Patients were operated on by the same team following the same dental program, and follow-up check-ups were carried out up to 12 months with a low loss of patients. A relevant contribution of our study was to explore the relationship between the questionnaire and clinical and therapeutic variables with a model of multiple linear regression analysis and correlation analysis. One of the limitations was the use of non-randomized control samples that may limit its external validity. Nor do we analyzed other factors that could influence OHRQOL, such as the type of cognitive deficit, bruxism, socioeconomic status, degree of collaboration, medication, and type of diet (4).

Conclusion

This is the first study conducted in Spain that provides data on OHRQOL in the mid-term in a group of mostly adults patients with ID after undergoing dental treatment under GA. Our findings illustrated that the overall score of the FHC-OHRQOL-Q and most of its dimensions improved significantly up to 12 months after treatment. The improvement of oral symptoms was significantly associated with DMFT index, decayed teeth, dental extractions and number of treatments. It can be inferred from the results of our study that the implementation of a dental treatment program in our clinical setting improved the OHRQOL in patients with ID.
